# Supplementary material for: Cross-cultural adaptation and psychometric validation of a Chinese self-intermittent catheterization quality of life scale among patients with neurogenic bladder
Source: PeerJ. 2024 Oct 24;12:e18319. doi: 10.7717/peerj.18319 (PMC11512800; doi:10.7717/peerj.18319)
Supplement: Supplemental Information 2 [file peerj-12-18319-s002.docx]

| Item | Strongly disagree | Slightly disagree | Neither agree nor disagree | Slightly agree | Strongly  agree |
| --- | --- | --- | --- | --- | --- |
| Ease of use | | | | | |
| 1.It is easy to prepare my catheter for use each time I need it |  |  |  |  |  |
| 2.It is messy to prepare my catheter for use |  |  |  |  |  |
| 3.I think it is easier to insert catheter |  |  |  |  |  |
| 4.Sometimes inserting catheter makes me feel uncomfortable |  |  |  |  |  |
| 5.The design of the catheter makes it easy to insert into the urethra |  |  |  |  |  |
| 6.I think it is inconvenient to use catheter |  |  |  |  |  |
| 7.The lubricant on the surface of the catheter makes it difficult to use |  |  |  |  |  |
| 8.I feel confident in my ability to use my catheter |  |  |  |  |  |
| Convenience | | | | | |
| 9.Storage of catheters at home is inconvenient |  |  |  |  |  |
| 10.It is not convenient to carry enough catheter when going out on weekends |  |  |  |  |  |
| 11.It is not convenient to carry enough catheters for two weeks of vacation |  |  |  |  |  |
| 12.Disposal of my catheter is inconvenient when away from home |  |  |  |  |  |
| Discreetness | | | | | |
| 13.It is easy to carry enough catheter around me every day |  |  |  |  |  |
| 14.I find it easy to dispose of my catheter when I am away from home |  |  |  |  |  |
| 15.My catheter is hidden |  |  |  |  |  |
| 16.When going out, I can use my catheter more covertly |  |  |  |  |  |
| 17.I can easily handle the catheter when others are not paying attention |  |  |  |  |  |
| 18.My catheter allows me to feel confident when away from home |  |  |  |  |  |
| Psychological well-being | | | | | |
| 19.I am self-conscious about my need to self-catherize |  |  |  |  |  |
| 20.I will feel embarrassed because others see my catheter or urine bag |  |  |  |  |  |
| 21.My need to use a catheter sometimes makes me feel embarrassed |  |  |  |  |  |
| 22.I worry that my catheter doesn’t always empty my bladder fully |  |  |  |  |  |
| 23.My need to use catheters stops me from visiting friends and family as often as I would like |  |  |  |  |  |
| 24.I am worried that using a catheter will cause long-term problems |  |  |  |  |  |
