# Supplementary material for: Cross-cultural adaptation and psychometric validation of a Chinese self-intermittent catheterization quality of life scale among patients with neurogenic bladder
Source: PeerJ. 2024 Oct 24;12:e18319. doi: 10.7717/peerj.18319 (PMC11512800; doi:10.7717/peerj.18319)
Supplement: Supplemental Information 3 [file peerj-12-18319-s003.docx]

| **量表翻译** |
| --- |
| **Ease of use** |
| 易用性 |
| 1.It is easy to prepare my catheter for use each time I need it |
| 每次需要导尿时我可以较容易地备好尿管 |
| 2. It is messy to prepare my catheter for use |
| 我认为准备（需使用的）尿管有些麻烦 |
| 1. I think it is easier to insert catheter |
| 我认为置入尿管较为容易 |
| 4. Sometimes inserting catheter makes me feel uncomfortable |
| 有时候插入尿管会让我感到不适 |
| 5. The design of the catheter makes it easy to insert into the urethra |
| 尿管的设计有利于其插入尿道 |
| 6. I think it is inconvenient to use catheter |
| 我认为使用尿管存在不便 |
| 7. The lubricant on the surface of the catheter makes it difficult to use |
| 尿管表面的润滑剂让尿管难以使用 |
| 8. I feel confident in my ability to use my catheter |
| 我对自己使用尿管的能力有信心 |
| **Convenience** |
| 便捷性 |
| 9. Storage of catheters at home is inconvenient |
| 在家中存放尿管较为不便 |
| 10. It is not convenient to carry enough catheter when going out on weekends |
| 携带足量尿管外出度过周末时较为不便 |
| 11. It is not convenient to carry enough catheters for two weeks of vacation |
| 携带足量尿管外出度过2周的假期较为不便 |
| 12. Disposal of my catheter is inconvenient when away from home |
| 出门在外时，处置尿管较为不便 |
| **Discreetness** |
| 隐蔽性 |
| 13. It is easy to carry enough catheter around me every day |
| 随身携带每日用量的尿管较为容易 |
| 14. I find it easy to dispose of my catheter when I am away from home |
| 出门在外时，处置尿管较为容易 |
| 15. My catheter is hidden |
| 我的尿管较为隐蔽 |
| 16. When going out, I can use my catheter more covertly |
| 出门在外时，我可以较为隐蔽地使用我的尿管 |
| 17. I can easily handle the catheter when others are not paying attention |
| 我能在别人不注意时轻而易举地处置尿管 |
| 18. My catheter allows me to feel confident when away from home |
| 携带尿管外出让我较为自信 |
| **Psychological well-being** |
| 心理健康 |
| 19. I am self-conscious about my need to self-catherize |
| 我意识到自己需要自行导尿 |
| 20. I will feel embarrassed because others see my catheter or urine bag |
| 我会因为别人看见我的尿管或尿袋而感觉尴尬 |
| 21. My need to use a catheter sometimes makes me feel embarrassed |
| 我有时会因使用尿管感到尴尬 |
| 1. I worry that my catheter doesn’t always empty my bladder fully |
| 我担心尿管无法每次都能将膀胱中的尿液排空 |
| 23. My need to use catheters stops me from visiting friends and family as often as I would like |
| 因为需要使用尿管，我无法频繁的拜访家人和朋友 |
| 24. I am worried that using a catheter will cause long-term problems |
| 我担心使用尿管会引发长期问题 |
